# Acute toxicity assessment of nine organic UV filters using a set of biotests

**DOI:** 10.1007/s43188-023-00192-2

**Published:** 2023-06-12

**Authors:** Stec Marcin, Astel Aleksander

**Affiliations:** Environmental Chemistry Research Unit, Institute of Biology and Earth Sciences, Pomeranian University in Słupsk, 22a Arciszewskiego Str., 76-200 Słupsk, Poland

**Keywords:** Acute toxicity, Biotest set, Environmental risk assessment, The toxicity of binary and ternary mixtures, UV filters

## Abstract

UV filters in environmental compartments are a source of concern related to their ecotoxicological effects. However, little is known about UV filters’ toxicity, particularly those released into the environment as mixtures. Acute toxicity of nine organic UV filters benzophenone-1, benzophenone-2, benzophenone-3, 4-methoxy benzylidene camphor, octocrylene, ethylhexyl methoxycinnamate, 2-ethylhexyl salicylate, homosalate, and butyl methoxydibenzoylmethane was determined. UV filter solutions were tested as single, binary, and ternary mixtures of various compositions. Single solutions were tested using a set of bio tests, including tests on saline crustaceans (*Artemia franciscana*), freshwater crustaceans (*Daphnia magna*), marine bacteria (*Aliivibrio fischeri*), and freshwater plants (*Lemna minor*). The tests represent different stages of the trophic chain, and hence their overall results could be used to risk assessment concerning various water reservoirs. The toxicity of binary and ternary mixtures was analyzed using the standardized Microtox^®^ method. Generally, organic UV filters were classified as acutely toxic. Octocrylene was the most toxic for *Arthemia franciscana* (LC_50_ = 0.55 mg L^–1^) and *Daphnia magna* (EC_50_ = 2.66–3.67 mg L^–1^). The most toxic against freshwater plants were homosalate (IC_50_ = 1.46 mg L^–1^) and octocrylene (IC_50_ = 1.95 mg L^–1^). Ethylhexyl methoxycinnamate (EC_50_ = 1.38–2.16 mg L^–1^) was the most toxic for marine bacteria. The least toxic for crustaceans and plants were benzophenone-1 (EC_50_ = 6.15–46.78 mg L^–1^) and benzophenone-2 (EC_50_ = 14.15–54.30 mg L^–1^), while 4-methoxy benzylidene camphor was the least toxic for marine bacteria (EC_50_ = 12.97–15.44 mg L^–1^). Individual species differ in their sensitivity to the tested organic UV filters. An assessment of the toxicity of mixtures indicates high and acute toxicity to marine bacteria after exposition to a binary mixture of benzophenone-2 with octocrylene, 2-ethylhexyl salicylate, or homosalate. The toxicity of mixtures was lower than single solutions predicting antagonistic interaction between chemicals.

**Graphical abstract:**

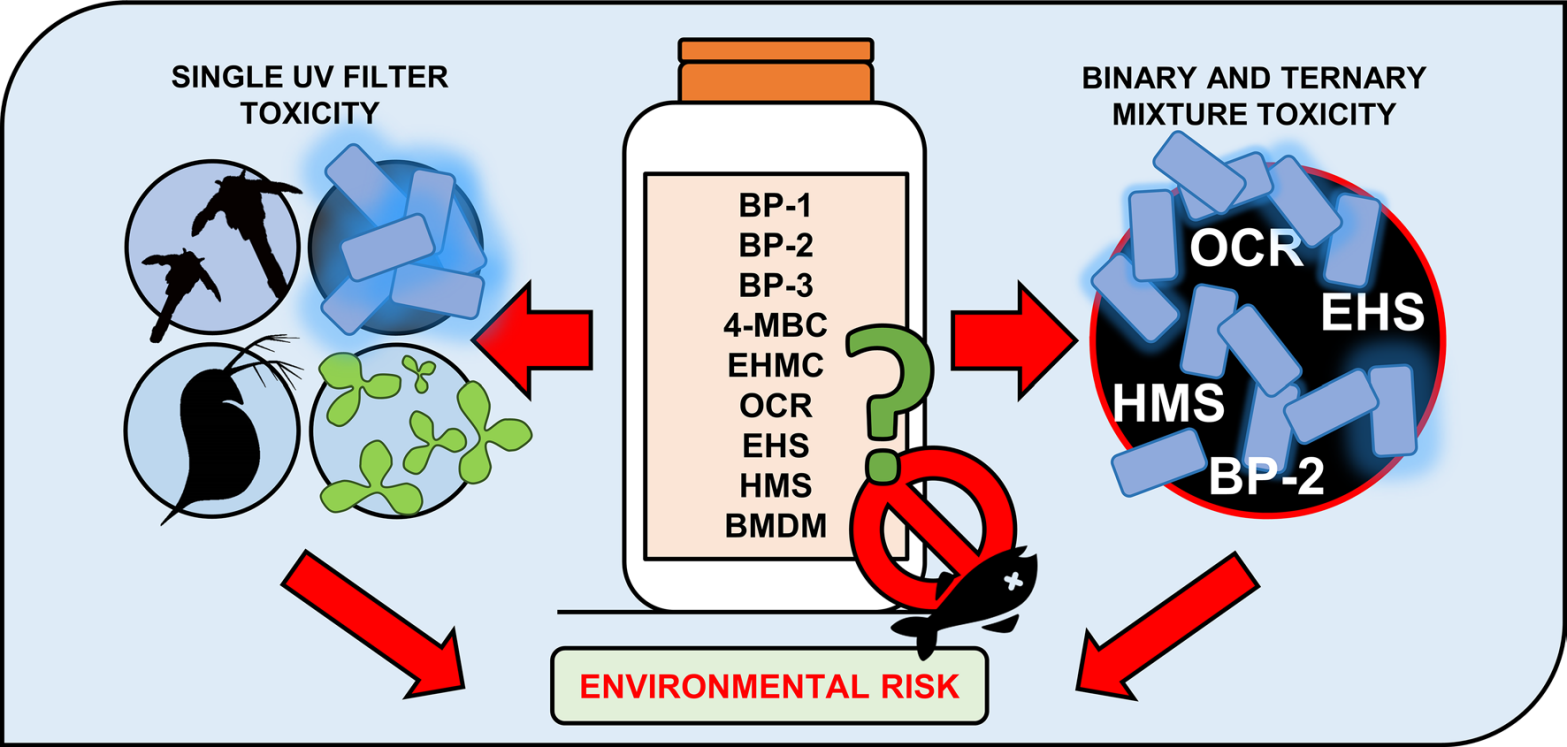

## Introduction

Water pollution is a worldwide problem that affects the biodiversity of living organisms and ecosystems. The number and variety of pollutants that are released into the environmental compartments due to natural processes and anthropogenic activity is a challenge for regulatory agencies. Although many contaminants are globally detectable, they are still not covered by integrated environmental monitoring. These pollutants are defined as emerging contaminants (ECs) and are categorized into several classes such as industrial chemicals, pesticides, repellents, plasticizers, pharmaceuticals, surfactants, and personal care products i.e. organic UV filters.

Since the 80s, education concerned with the negative impacts of excessive sun exposure and the importance of sunscreen usage has promoted a developing market for sunscreen products. Currently, UV filters are ingredients of sunscreen agents and daily-use products. According to estimations, in 1998 worldwide market of sunscreen products generated profits of 521 million USD. The estimated amount of UV filters present in these cosmetics was about 9.2 million tons. This is consistent with recent estimations that approximately 10 million tons of UV filters are produced annually on the global market. Current data and forecasts in the global sunscreen market indicate, that the demand for sunscreen products continuously increases. In 2022, the global market for sunscreen products was estimated to have generated revenue of 10.3 billion USD and is expected to reach USD 13.64 billion USD in 2026 [1].

The widespread daily use of UV filters has contributed to their presence in the aquatic environment. UV filters are released into the environment through direct sources such as coastal tourism and recreational activities in water [2–4]. It is estimated that around 25% of sunscreen applied to the skin is washed off in a single bath [5, 6]. Indirect sources of UV filters are insufficiently treated effluents from wastewater treatment plants (WWTPs) [7–9]. BP-1 (benzophenone-1), BP-2 (benzophenone-2), BP-3 (benzophenone-3), 4-MBC.

(4-methoxybenzylidene camphor), OCR (octocrylene), EHMC (ethylhexyl methoxycinnamate), EHS (2-ethylhexyl salicylate), HMS (homosalate), and BMDM (butyl methoxydibenzoylmethane) were detected in different environmental matrices such as freshwater (< LOD–4381 ng L^–1^) [10–14], seawater (< LOD– 172 µg L^–1^) [4, 15–20], beach sediments [21–23], sea and river sediments [24, 25]. UV filters were found in the seawater and sediments near crowded parts of the coast [4, 17, 20–22], as well as in coastal protected areas [21, 26], and even in regions with low or negligible anthropogenic pressure such as the Antarctic [16, 27]. Currently, organic UV filters are identified as emerging contaminants of one of the highest environmental priorities.

Reports prove that many organic UV filters are not biodegradable and accumulate in the environment [28–30]. Limited biodegradation is due to the value of the octanol/water partition coefficient (Log K_ow_). A value of Log K_ow_ > 1 characterizes substances easily soluble in fatty solvents, while the value of Log K_ow_ < 1 is typical for hydrophilic molecules [31]. Most of the organic UV filters tend to values of Log K_ow_ > 4 demonstrating their ability to bioaccumulate in living organisms, and hence different chains of the trophic food webs. UV filters were reported in mussels (2–7112 ng g^−1^ d.w.) [32], crustaceans (< 10–68.9 ng g^−1^ d.w.) [33], various species of marine and freshwater fish (< LOD-680 ng g^−1^ d.w.) [34, 35], and in sea mammals (89–782 ng g^−1^ l.w.) [36]. Fent et al. [37] monitored the widespread occurrence of UV filters at different trophic levels indicating the bioaccumulation of EHMC in the food chain. Detected concentrations ranged between 22 and 150 ng g^−1^ l.w. in mussels (*Dreissena polymorpha*), between 16 and 701 g^−1^ l.w. in cormorants (*Phalacrocorax* sp.), and around 337 g^−1^ l.w. in barb (*Barbus barbus*) [37].

There are growing concerns about the safety and potential toxicity of UV filters. UV filters were toxic against several species used for toxicological testing including marine bacteria [38], algae [39–41], corals [5, 42, 43], crustaceans [41, 44], and fish [45–48]. It has been observed that organic UV filters contribute to slowing down the photosynthesis and growth of green algae [39, 41, 49], coral reef bleaching [5, 50–52], show toxic effects for embryos, and cause defects in juveniles organisms [47, 48, 53, 54]. Moreover, some of the organic UV filters are capable of disrupting endocrine systems. Published reports indicate their contribution to the reduction of fertility and reproduction [55–58] and prove to have estrogenic activity [45, 58–61].

Biotests are the most popular and low-cost method for assessment of the ecotoxicological risk related to ECs released into the environment [62–64]. Although the list of organic UV filters approved for use in the European Union includes about 30 substances [65], most ecotoxicological studies concern only BP-3, EHMC, and 4-MBC [37, 43, 66]. Recently, more studies take into account the toxicity of other commonly used organic UV filters, such as OCR, BMDM, EHS, and HMS [41, 67, 68]. However, the toxic effect of UV filters on various organisms is not fully understood [69] and still requires some new clues and pieces of evidence. Additionally, ecotoxicological studies on organic UV filters most often prioritize an assessment of toxicity on organisms from higher trophic levels such as fish [34, 35, 37, 61, 70], mammals [36], and birds [37, 71].

As the sensitivity and response of single biotests or living organisms to chemical contaminants vary widely, the biotest sets with different sensitivity profiles should be used for the comprehensive assessment of the ecological risk. Ecotoxicological hazard predictions are more informative if the biotest sets involve organisms of different trophic levels. Any disorders in populations from lower trophic levels can result in a chain reaction at higher levels. Finally, toxic effects found for simple bacteria or primary reducents disrupt the functioning of the entire ecosystem [38, 42]. Moreover, in the given biocenosis, organisms are exposed to mixtures of different UV filters which interact with each other [47]. Mixtures of UV filters can be more toxic than single substances or show a reduced toxic effect. However, a detailed literature survey indicates there is a lack of clear information covering the toxicity interactions and their effects are poorly understood [72].

Therefore, this study aimed to assess the toxicity of nine organic UV filters using standardized organisms commonly used in ecotoxicity tests that represent marine and freshwater ecosystems. The main objectives were: (i) to investigate the acute toxicity of a single tested organic UV filter using a set of four biotests; (ii) to categorize toxicity according to established classifications; (iii) to investigate the toxicity of the binary and ternary mixtures of selected UV filters. Standard toxicity measures (EC_50_, LC_50_, IC_50_) were used to classify their independent toxicity.

## Materials and methods

### Preparation of stock and working solutions

Analytical standards (purity > 98%) of each organic UV filter, as well as organic solvents (methanol (MeOH) and dimethyl sulfoxide (DMSO) of purity > 99%), were purchased from Merck (Germany). The main characteristics of the tested organic UV filters were summarized in Table 1 [20, 34].

**Table 1:**
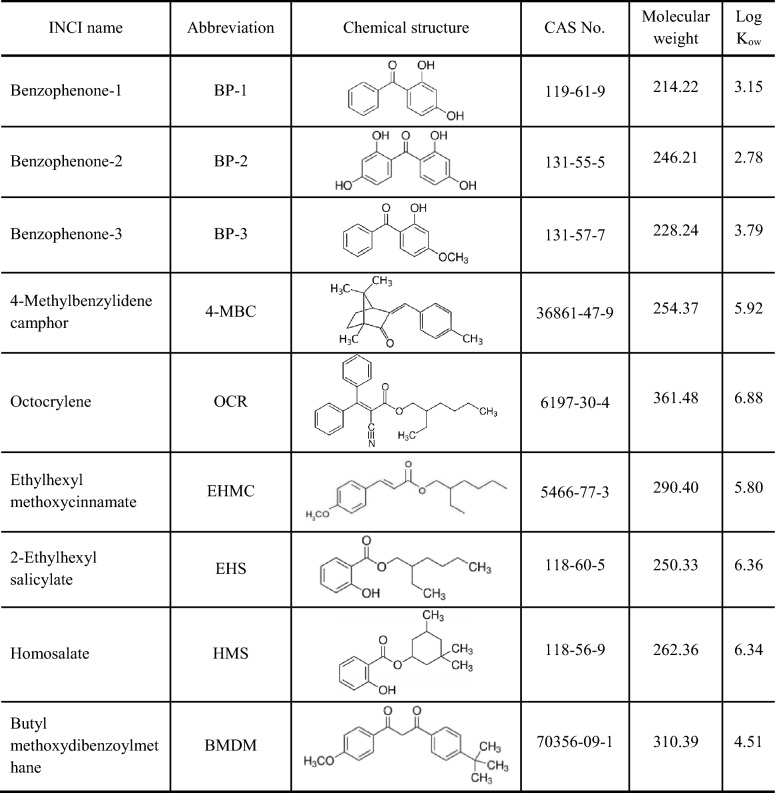
Main characteristics of the tested UV filters

Based on literature data MeOH [49, 73, 74] and DMSO [41, 68, 75] were preselected as necessary additives for the stock solutions since UV filters are generally characterized by limited solubility in deionized water, as well as in the test growth medium. Before the stock solutions were prepared, the toxicity of MeOH and DMSO was primarily determined. Finally, DMSO was selected for the study, since at 1% concentration it did not cause any test reaction. A series of dilutions and control of each test were prepared using 1% DMSO. Basic concentrations of each UV filter were prepared according to previous recommendations [41, 68, 71, 73, 75]. An initial concentration of all UV filters used in the Microtox® screening test was 10 mg L^–1^. For the rest of the tests (Artoxkit M™, Daphtoxkit^FT^, *Lemna* sp. GIT), an initial concentration was 100 mg L^–1^ (BP-1, BP-2), 12.5 mg L^–1^ ( BP-3) and 10 mg L^–1^ (OCR, EHMC, EHS, HMS, BMDM).

### Toxicity tests procedures

The acute toxicity of the standard solutions of nine organic UV filters was assessed by the dilution test method using four standardized test organisms: marine bacteria *Aliivibrio fischeri* (Microtox^®^), brine shrimp *Artemia franciscana* (Artoxkit M™), freshwater crustaceans *Daphnia magna* (DaphtoxkitF™), and aquatic plant *Lemna minor* (*Lemna sp.* GIT). Tests were performed according to individual standard procedures. Tested organisms (except *Lemna minor*), and all necessary consumables were purchased from Tigret (Warsaw, Poland).

### Marine bacteria *Aliivibrio fischeri* luminescence inhibition test

The test was conducted once according to ISO 11348–3 standard [76]. MicrotoxOmni™ Screening Test was carried out using a screening test (81.9% Screening Test), followed by a dilution test (81.9% Basic Test) in the Microtox Model 500 analyzer (Modern Water Inc., New Castle, DE, USA) that operates both as an incubator and as a photometer. The percentage of bioluminescence inhibition was measured after 5, 15, and 30 min exposition to organic UV filter solutions relative to the control sample. For each exposition time, an effective concentration causing inhibition luminescence on 50% of bacteria (EC_50_) was determined. The analogical procedure of the MicrotoxOmni™ Screening Test was used by others [77, 78].

### Marine crustacean *Artemia franciscana* mortality test

The test was carried out on freshly hatched saline crustaceans *Arthemia franciscana* according to ASTM Standard Guide E1440-91 [79]. The cysts of brine shrimps were incubated at 25 °C for 22 h in a seawater medium under constant lighting. Freshly hatched crustaceans (< 24 h) were selected through a light source. Three sets of 10 organisms in each, were transferred into multi-well test plates filled with 1 ml solution of organic UV filter per well. None of the mortality of the test organisms was detected after 24 h exposition. However, some behavioral change was observed, and this is why, after 24 h exposition time an EC_50_ was determined. A lack of mortality after 24 h exposition convinced us to increase the exposition time to 48 h, and then the number of dead organisms was counted, and hence the lethal concentration (LC_50_) was estimated. After the test was accomplished the behavioral effects observed or the number of dead organisms counted in three wells were averaged, and the corresponding standard deviation was calculated. A similar procedure was applied by others [41, 68, 78]. To exclude the possible effect of mortality due to lack of food, test organisms were also incubated in DMSO and the corresponding check was done after 24 h and 48 h. A check has proved the satisfactory condition of the tested organisms. None of the mortality was discovered in control samples.

### Freshwater crustacean *Daphnia magna* immobilization test

The test was carried out on freshly hatched crustaceans *Daphnia magna* from the surviving eggs, according to OECD Guideline 202 standard [80]. Tested organisms were incubated at 20 °C for 72 h in the freshwater medium under constant lighting. Before the experiment, crustaceans were fed with powdered spirulina. Freshly hatched crustaceans were selected through a light source. Three sets of 5 tested organisms in each, were transferred into multi-well test plates filled with 10 ml of organic UV filter solution per well. Each solution was tested in triplicate. Immobilized crustaceans were counted in each of the three wells after 24 and 48 h of exposition, and the corresponding average and standard deviation was computed. After the experiment was accomplished EC_50_ values were estimated. The analogical procedure of the DaphtoxkitF™ test was applied by others [77, 81, 82].

### Lemna minor growth inhibition test

*Lemna minor* growth inhibition test (*Lemna sp.* GIT) was performed according to OECD Guideline 221 standard [83]. Tested plants cultivated in controlled laboratory conditions were used for the tests. Plants were grown on sterile Steinberg's medium at 25 °C under constant lighting. One plant with 2 or 3 fronts was introduced into the well of the test plate filled with 10 ml of organic UV filter solution per well. The test for every concentration was done in triplicate. On 1st day and 7th day images of the test plates were acquired. Based on the results obtained by measuring the leaf area, an inhibitory concentration (IC_50_) that inhibits *Lemna minor* growth was estimated. The analogical protocol of the *Lemna *sp. GIT test was used by others [81–83].

### Binary and ternary mixtures toxicity assessment

To assess a type of ecological risk caused by the release of UV filter mixtures into the environment a recommended, bioluminescence inhibition test was used. The MicrotoxOmni™ Screening Test is very often used as a first test of the set of biotests [84, 85] and is highly preferable as a rapid toxicity testing system by the US Environmental Protection Agency [86]. The toxicity of two-component (binary) and three-component (ternary) mixtures of UV filters was experimentally tested. Because the total number of different binary combinations was 21 and the total number of ternary combinations exceeded 120 we subjectively decided to decrease the number of tested mixtures to 13 combinations of binary and 6 combinations of ternary mixtures with benzophenones as the dominant base due to the limited access to consumables*.* The selection of benzophenones as one of the components was because they are one of the most frequently UV filters determined in the environmental compartments and some pieces of evidence concerning their toxic effects in binary mixtures on the *Chironomus riparius* are already available [87]. Observed adverse effects of benzophenones include disruption of the endocrine system of organisms, effects on reproduction, developmental toxicity, and neurotoxicity [88]. All tests were conducted using the protocol of the MicrotoxOmni™ Screening Test described above [76]. Every mixture was tested once. The initial concentration of each tested UV filter in the mixture was 10 mg L^−1^. Components of the mixture were assigned in ratio 1:1 or 1:1:1, respectively. Mixtures toxicity was estimated using EC_50_ (%). Since the toxicity of the mixture can be different in comparison with the toxicity of a single solution due to the interaction between chemicals, the predicted type of toxic interactions in mixtures was classified according to Park et al. [44], while the toxicity units (TU_mix_) were mathematically estimated using the formula:

1 $${{\varvec{T}}{\varvec{U}}}_{{\varvec{m}}{\varvec{i}}{\varvec{x}}}=\sum _{i=1}^{n}\frac{{\mathbf{C}}_{\mathbf{i}}}{{\mathbf{E}\mathbf{C}}_{50\mathbf{i}}}$$

where: C_i_ is the concentration of ith UV filter in the tested mixture, EC_50i_ is the individual effective concentration for a given UV filter, and *n* stands for the number of filters in the mixture. A total TU_mix_ value less than 1 indicates synergic toxicity, equal to 1 expresses additive toxicity, while a value greater than 1 is for antagonistic effect.

### Software and toxicity classification approach

All graphs were prepared using Excel (Microsoft, Palo Alto, CA, USA). The estimated values of EC_50_, LC_50,_ and IC_50_ were determined using linear and logarithmic regressions calculated for the effect-dose relationship. An agreement between observed and predicted effects was assessed based on the determination coefficient (R^2^) value (p = 0.05) calculated using R-Pearson [89] and the correlation coefficient for logarithmic regressions [90], respectively. Results of the Microtox^®^ test were automatically generated, using the MicrotoxOmni™ software, as reports including an EC_50_ parameter, while in the case of the test with *Lemna minor,* the analysis of recorded images was accomplished using the software ImageJ v 1.51w (release available at https://imagej.nih.gov/ij/all-notes.html).

Estimated EC_50_, LC_50,_ and IC_50_ values were used to assess and classify an environmental risk caused by UV filters. Toxicity classification concerning single biotests and mixtures was done according to criteria proposed by Persoone et al. [91] who specify toxicity classes according to EC_50_ expressed in % or EC_50_, LC_50_, and IC_50_ expressed in mg L^–1^ as follows: very high acute toxicity (-%; > 0.1 mg L^–1^), high acute toxicity (> 25%; 0.1**–**1 mg L^–1^), acute toxicity (25–75%; 1**–**10 mg L^–1^), low acute toxicity (75–100%; 10**–**100 mg L^–1^), and non-toxic (< 100%; < 100 mg L^–1^).

## Results

### Marine bacteria luminescence inhibition

**Fig. 1:**
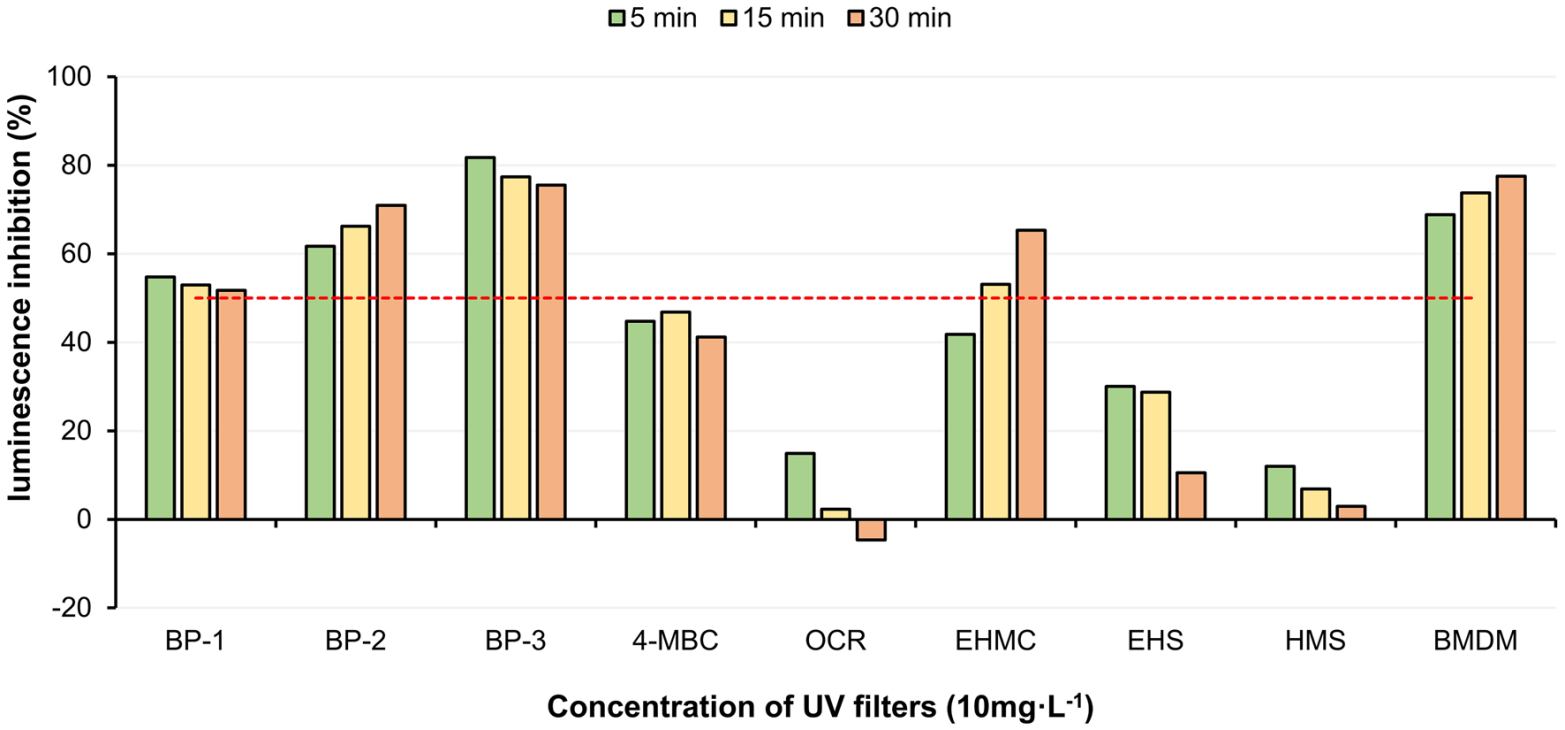
Bioluminescence inhibition for single UV filters in the Microtox® screening test

In the *Aliivibrio fischeri* luminescence inhibition test, the screening showed that BP-1, BP-2, BP-3, EHMC, and BMDM were characterized by toxicity effects higher than 50% (Fig. 1). Bacteria exposed to OCR, EHS, and HMS solutions did not show a 50% decrease in luminescence inhibition.

The toxicity of the organic UV filters to *Aliivibrio fischeri* was different, with the following downward trends observed: (i) BP-3 > BMDM > BP-2 > BP-1 > 4-MBC > EHMC, (ii) BP-3 > BMDM > BP-2 > EHMC > BP-1 > 4-MBC, (iii) BMDM > BP-3 > BP-2 > EHMC > BP-1 > 4-MBC after 5, 15 and 30 min exposition, respectively.

**Fig. 2:**
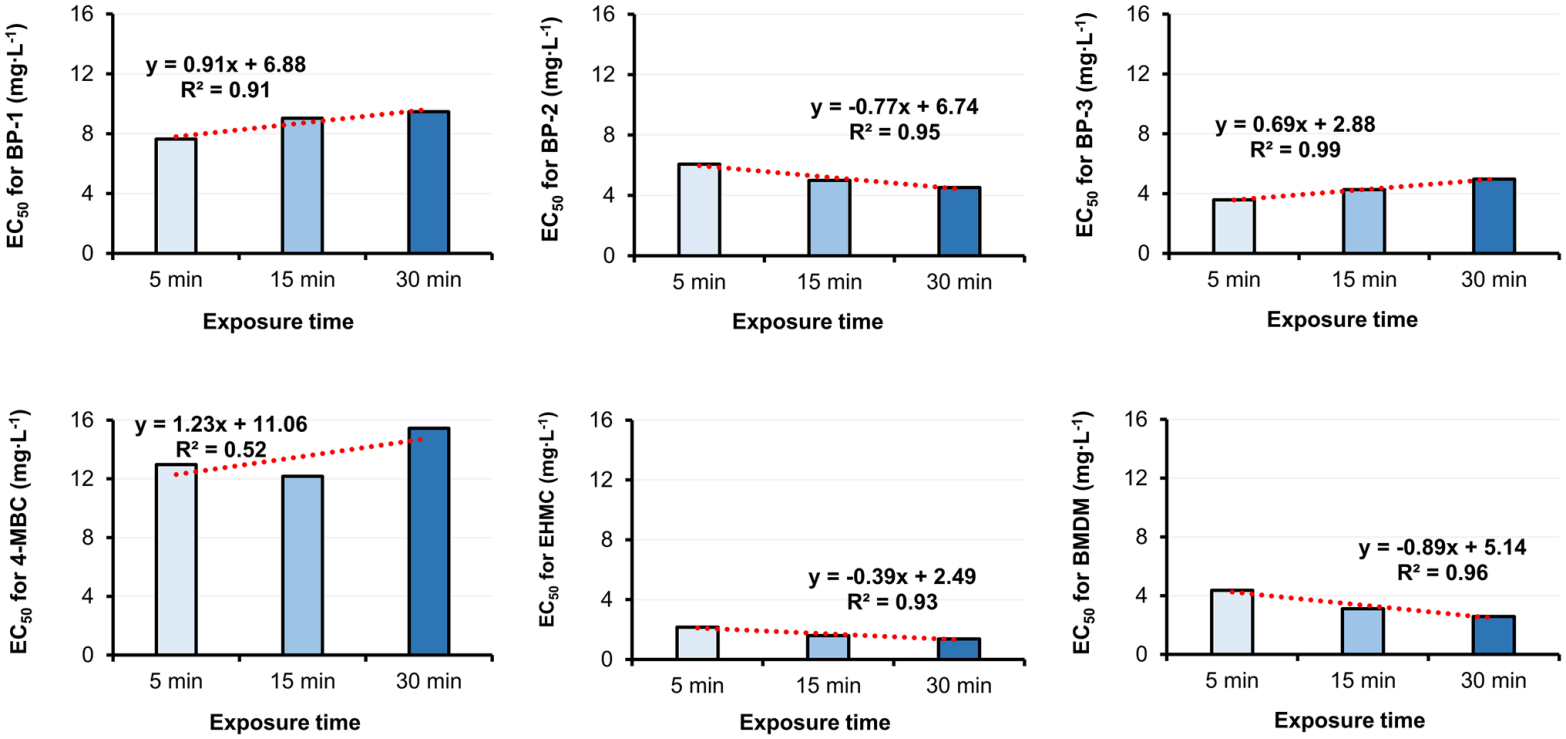
Estimated EC_50_ values obtained for single UV filters in the Microtox® basic test

Consecutively, BP-1, BP-2, BP-3, and EHMC were further tested to estimate EC_50_ value using the dilution procedure. 4-MBC has shown around 50% luminescence inhibition after 15 min, and this is why it was also qualified for further evaluation. Moreover, to analyze the dynamics of toxicity effect over time the linear regression and determination coefficient for luminescence inhibition as the dependent variable and exposure time as an independent variable for BP-1, BP-2, BP-3, 4-MBC, EHMC, and BMDM were computed (Fig. 2).

An increase in the toxic effect for BP-2, EHMC, and BMDM was observed over time, while for BP-1 and BP-3 an opposite effect was discovered. Except for 4-MBC, all linear regressions were characterized by a highly significant determination coefficient (R^2^_computed_: 0.91 (BP-1) – 0.99 (BP-3), R^2^_crticial_: 0.77 (n = 3, p = 0.05)). The quantitative assessment indicates a 60% decrease in EC_50_ value over time for EHMC, BMDM, and around 40% for BP-2. As presented above, only for some compounds, the toxicity decrease as the exposure time increases. Since every step of the screening procedure was accomplished in agreement with the test protocol an opposite toxicity change over time was rather not caused by the instability of the data due to a small number of samples. It suggests that marine bacteria can adapt to the presence of some xenobiotics.

### Marine crustacean mortality and freshwater crustacean immobilization

**Fig. 3:**
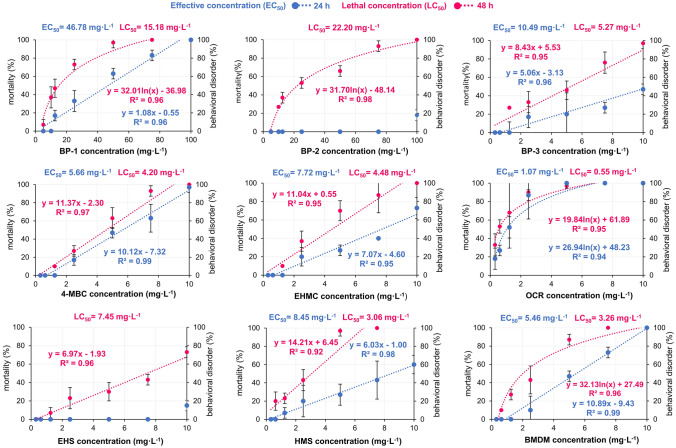
Estimated EC_50_ and LC_50_ values in the Artoxkit M™ test

Although saline and freshwater crustaceans *Artemia franciscana* and *Daphnia magna*, respectively, are important in feeding early life-cycle stages of marine and freshwater fish, literature data for the toxicity of many UV filters on these crustaceans are ambiguous [44, 92, 93]. In the current research, none of the toxic effects was observed after 24 h *Artemia franciscana* exposition to BP-2, and EHS. For seven of nine UV filters, behavioral changes manifested in reverse motion were observed, enabling estimation of EC_50_ after 24 h exposition. After 48 h crustacean mortality for nine tested UV filters was observed, enabling the estimation of LC_50_. Linear and logarithmic response as well as calculated EC_50_ and LC_50_ values for the Artoxkit M™ test are depicted in Fig. 3.

After 48 h exposure, LC_50_ values were in the range of 0.55–22.20 mg L^–1^. The lowest toxic concentration was estimated for OCR (0.55 mg L^–1^) followed by HMS (3.06 mg L^–1^), and BMDM (3.26 mg L^–1^). Among benzophenones, the most toxic was BP-3 (5.27 mg^.^L^–1^). BP-2 and BP-1 were the least toxic with a concentration of 22.20 mg^.^L^–1^ and 15.18 mg^.^L ^–1^, respectively.

**Fig. 4:**
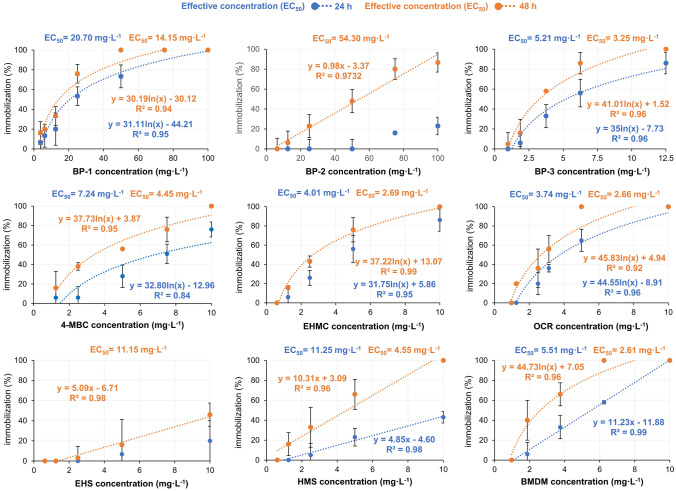
Estimated EC_50_ values in the Daphtoxkit F™ test

Similarly, as in the case of *Artemia franciscana*, none of the toxic effects was observed after 24 h *Daphnia magna* exposition to BP-2 and EHS. For seven of nine, and all of the tested filters reverse motion and immobilization were observed after 24 h and 48 h exposition, respectively. Linear and logarithmic responses as well as calculated EC_50_ and LC_50_ values for the Daphtoxkit F™ test are depicted in Fig. 4.

EC_50_ values after 48 h exposure were in the range of 2.61–54.30 mg L^–1^. The lowest EC_50_ was estimated for BMDM (2.61 mg L^–1^), OCR (2.66 mg L^–1^), and EHMC (2.69 mg L^–1^), respectively. Among the benzophenone-type UV filters, BP-3 was highly toxic (3.25 mg L^–1^), while BP-2 was the least toxic (54.30 mg L^–1^). Similar toxic concentration values were reported by Park et al. [44] for corresponding filters: BMDM—1.95 mg L^–1^, OCR—3.18 mg L^–1^, and EHMC—2.73 mg L^–1^. However, of the three UV filters tested by Park et al. [44], OCR was the least toxic. The toxic concentration observed for BP-2 (48.26 mg L^–1^) by Liu et al. [75] was similar to that observed in the current research. According to toxicity classification classes, in the test with *Daphnia magna*, seven of nine filters were classified as compounds of acute toxicity. BP-1 and BP-2 were the least toxic and classified as molecules of low acute toxicity. An equivocal assessment of UV filter toxicity against *Daphnia magna* is not a trivial task since a set of significantly lower toxic concentrations was also reported in references. Contrary to current and referenced results, Boyd et al. [67] reported significantly lower toxic concentrations of OCR, BP-3, and EHMC to *Daphnia magna*. In their work, the 48 h EC_50_ values were 0.03 mg L^–1^ for OCR and 1.2 mg L^–1^ for BMDM and BP-3, respectively. Another set of results presented by de Paula et al. [67] indicates that the most toxic for *Daphnia magna* was EHMC (0.50 mg L^–1^) followed by BP-3 (1.73 mg L^–1^), BMDM (1.89 mg L^–1^) and OCR (2.97 mg L^–1^). Kudlek and Dudziak [80] observed toxic concentrations of BP-3 in the range of 2.0–2.53 mg L^–1^.

### Aquatic plant *Lemna minor* growth inhibition

**Fig. 5:**
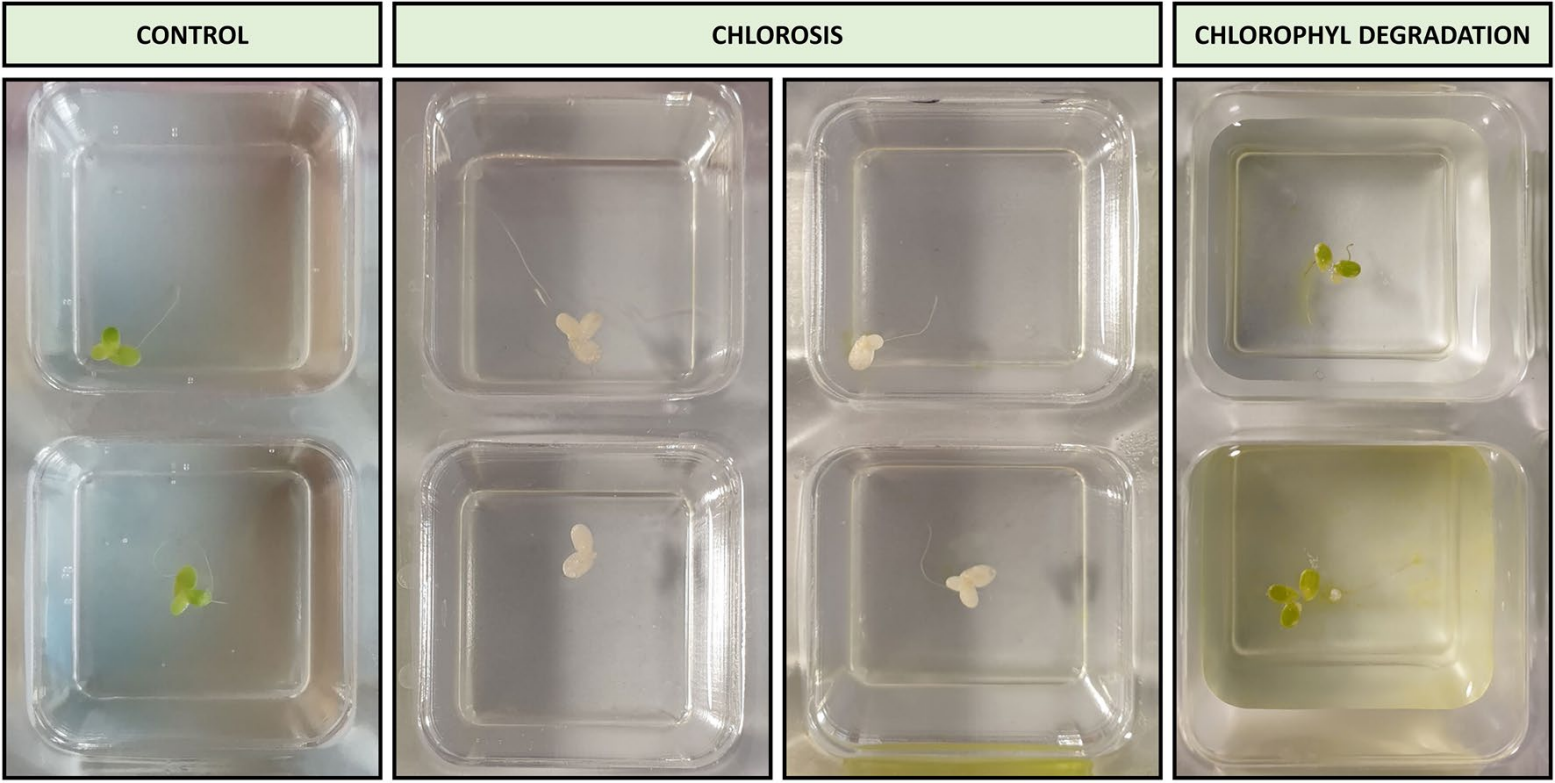
Visible defects *Lemna minor* after exposition to UV filters in comparison to control

**Fig. 6:**
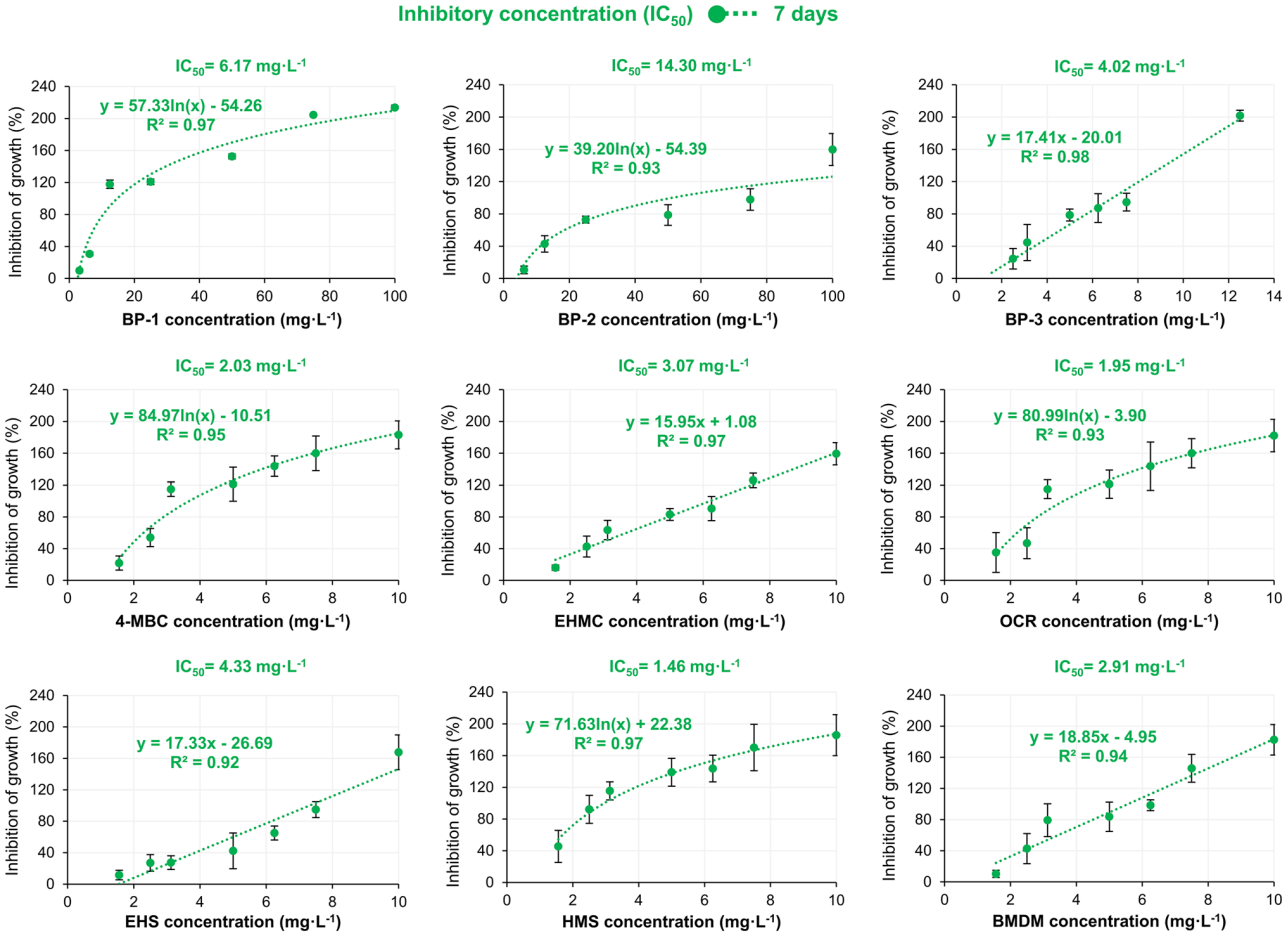
Estimated IC_50_ values in the *Lemna *sp. GIT test after 7 days exposition

All tested UV filters induced a significant decrease in frond number and frond area in the freshwater plant *Lemna minor* in test *Lemna *sp. GIT after 7 days exposition. Other visible defects, such as changes in the color of the frond, were additionally observed (Fig. 5). The calculated IC_50_ values for organic UV filters against *Lemna minor* plant are shown in Fig. 6.

Generally, there was not any increase observed in the frond area after *Lemna minor* exposition to all tested UV filters. The lowest growth inhibition concentration was estimated for HMS (1.46 mg L^–1^) and OCR (1.95 mg L^–1^), followed by 4-MBC (2.03 mg L^–1^), BMDM (2.91 mg L^–1^), and EHMC (3.07 mg L^–1^). The least toxicity was estimated for BP-2 with IC_50_ value 14.30 mg L^–1^.

### Toxicity of mixtures

The toxicity of the binary and ternary mixtures was accessed by the Microtox® test for ecological risk assessment and verification. The calculated EC_50_ values (%) are summarized in Table 2.

**Table 2:** Calculated EC_50_ (%) values of binary and ternary mixtures of UV filters

Mixture	EC_50_ (%)			Toxicity class
	5 min	15 min	30 min	
BP-1 + BP-2	49.54	47.61	39.22	Acute toxicity
BP-1 + 4-MBC	< 100	< 100	< 100	Non-toxic
BP-1 + EHMC	60.72	69.80	72.91	Acute toxicity
BP-1 + BMDM	61.19	64.59	88.26	Acute toxicity/low acute toxicity
BP-2 + BP-3	29.88	32.78	46.04	Acute toxicity
BP-2 + 4-MBC	< 100	< 100	< 100	Non-toxic
BP-2 + EHMC	1.0	1.09	0.91	High acute toxicity
BP-2 + BMDM	< 100	< 100	< 100	Non-toxic
BP-2 + OCR	0.56	0.63	0.67	High acute toxicity
BP-2 + EHS	0.88	0.94	1.32	High acute toxicity
BP-2 + HMS	1.32	2.56	3.04	High acute toxicity
BP-3 + EHMC	1.04	1.06	2.29	High acute toxicity
BP-3 + BMDM	41.81	42.91	96.11	Acute toxicity /low acute toxicity
4-MBC + EHMC	44.61	51.58	47.26	Acute toxicity
BP-1 + BP-2 + BP-3	44.23	46.18	65.30	Acute toxicity
BP-2 + BMDM + EHMC	< 100	80.91	73.01	Non-toxic/acute toxicity
BP-1 + BP-3 + EHMC	< 100	< 100	< 100	Non-toxic
BP-2 + BP-3 + EHMC	2.48	1.41	1.56	High acute toxicity
BP-2 + BP-3 + BMDM	71.46	66.24	59.15	Acute toxicity
BP-2 + EHS + HMS	4.61	1.73	0.88	High acute toxicity

Results indicate that 3/4 of the EC_50_ values were below 73% (mean 46%). Most of the mixtures are classified as high and acutely toxic for marine bacteria *Aliivibrio fischeri*. The majority of the most toxic binary and ternary mixtures possessed EHMC as a compound. In the set of EHMC-based mixtures following descending toxicity trend was observed: BP-2 + EHMC > BP-2 + BP-3 + EHMC > BP-3 + EHMC > 4-MBC + EHMC > BP-1 + EHMC > BP-2 + BMDM + EHMC. Mixtures were characterized by EC_50_ values in the range between 0.91 and 73.01% after 30 min exposition. In the case of ternary mixtures based on BP-2, the lowest toxic concentration was observed for a combination of BP-2 + EHS + HMS and BP-2 + BP-3 + EHMC, characterized by EC_50_ values of 0.88% and 1.56%, respectively. Generally, the highest number of non-toxic mixtures that did not trigger a strong response after 5 min exposition contained BP-2 as one of the ingredients. Mixtures containing a combination of BP-2 + 4-MBC, BP-2 + BMDM, and BP-2 + BMDM + EHMC were classified as non-toxic (EC_50_ > 100%) after 5 min exposition, however, in the last case, the toxicity slightly increased over exposition time. Surprisingly, single UV filters (OCR, EHS, HMS) that were classified as not toxic in MicrotoxOmni™ Screening Test become highly toxic in conjunction with BP-2.

Table 3 contains summarized TU_mix_ values. TU_mix_ values can be understood as the type of toxicity interaction prediction using EC_50_ values for a single solution obtained in the MicrotoxOmni™ Screening Test after 30 min exposition while in Table 4 toxicity classes for each UV filter according to the type of tested organism (freshwater or saltwater) were summarized.

**Table 3:** Estimated TU_mix_ values and predicted type of toxicity interaction

Mixture	TU_mix_ calculation after 30 min	TU_mix_	Predicted toxicity interaction type
BP-1 + BP-2	$\frac{{10\; {\varvec{m}}{\varvec{g}}\cdot {\varvec{L}}}^{-1}}{{9.47 {\varvec{m}}{\varvec{g}}\cdot {\varvec{L}}}^{-1}}$+$\frac{{10\; {\varvec{m}}{\varvec{g}}\cdot {\varvec{L}}}^{-1}}{{4.52 {\varvec{m}}{\varvec{g}}\cdot {\varvec{L}}}^{-1}}$	3.27	Antagonism
BP-1 + 4-MBC	$\frac{{10\; {\varvec{m}}{\varvec{g}}\cdot {\varvec{L}}}^{-1}}{{9.47 {\varvec{m}}{\varvec{g}}\cdot {\varvec{L}}}^{-1}}$+$\frac{{10\; {\varvec{m}}{\varvec{g}}\cdot {\varvec{L}}}^{-1}}{{15.44 {\varvec{m}}{\varvec{g}}\cdot {\varvec{L}}}^{-1}}$	1.71	Antagonism
BP-1 + EHMC	$\frac{{10\; {\varvec{m}}{\varvec{g}}\cdot {\varvec{L}}}^{-1}}{{9.47 {\varvec{m}}{\varvec{g}}\cdot {\varvec{L}}}^{-1}}$+$\frac{{10\; {\varvec{m}}{\varvec{g}}\cdot {\varvec{L}}}^{-1}}{1.38{\boldsymbol{ }{\varvec{m}}{\varvec{g}}\cdot {\varvec{L}}}^{-1}}$	8.31	Antagonism
BP-1 + BMDM	$\frac{{10\; {\varvec{m}}{\varvec{g}}\cdot {\varvec{L}}}^{-1}}{{9.47 {\varvec{m}}{\varvec{g}}\cdot {\varvec{L}}}^{-1}}$+$\frac{{10\; {\varvec{m}}{\varvec{g}}\cdot {\varvec{L}}}^{-1}}{2.58 {{\varvec{m}}{\varvec{g}}\cdot {\varvec{L}}}^{-1}}$	4.93	Antagonism
BP-2 + BP-3	$\frac{{10\; {\varvec{m}}{\varvec{g}}\cdot {\varvec{L}}}^{-1}}{{4.52 {\varvec{m}}{\varvec{g}}\cdot {\varvec{L}}}^{-1}}$+$\frac{{10\; {\varvec{m}}{\varvec{g}}\cdot {\varvec{L}}}^{-1}}{4{.97 {\varvec{m}}{\varvec{g}}\cdot {\varvec{L}}}^{-1}}$	4.22	Antagonism
BP-2 + 4-MBC	$\frac{{10\; {\varvec{m}}{\varvec{g}}\cdot {\varvec{L}}}^{-1}}{{4.52 {\varvec{m}}{\varvec{g}}\cdot {\varvec{L}}}^{-1}}$+$\frac{{10\; {\varvec{m}}{\varvec{g}}\cdot {\varvec{L}}}^{-1}}{{15.44 {\varvec{m}}{\varvec{g}}\cdot {\varvec{L}}}^{-1}}$	2.86	Antagonism
BP-2 + EHMC	$\frac{{10\; {\varvec{m}}{\varvec{g}}\cdot {\varvec{L}}}^{-1}}{{4.52 {\varvec{m}}{\varvec{g}}\cdot {\varvec{L}}}^{-1}}$+$\frac{{10\; {\varvec{m}}{\varvec{g}}\cdot {\varvec{L}}}^{-1}}{1.38{\boldsymbol{ }{\varvec{m}}{\varvec{g}}\cdot {\varvec{L}}}^{-1}}$	9.46	Antagonism
BP-2 + BMDM	$\frac{{10\; {\varvec{m}}{\varvec{g}}\cdot {\varvec{L}}}^{-1}}{{4.52 {\varvec{m}}{\varvec{g}}\cdot {\varvec{L}}}^{-1}}$+$\frac{{10\; {\varvec{m}}{\varvec{g}}\cdot {\varvec{L}}}^{-1}}{2.58 {{\varvec{m}}{\varvec{g}}\cdot {\varvec{L}}}^{-1}}$	6.09	Antagonism
BP-2 + OCR	Not calculated	Not calculated	Not estimated
BP-2 + EHS	Not calculated	Not calculated	Not estimated
BP-2 + HMS	Not calculated	Not calculated	Not estimated
BP-3 + EHMC	$\frac{{10\; {\varvec{m}}{\varvec{g}}\cdot {\varvec{L}}}^{-1}}{{4.97\; {\varvec{m}}{\varvec{g}}\cdot {\varvec{L}}}^{-1}}$+$\frac{{10\; {\varvec{m}}{\varvec{g}}\cdot {\varvec{L}}}^{-1}}{1.38 {{\varvec{m}}{\varvec{g}}\cdot {\varvec{L}}}^{-1}}$	9.26	Antagonism
BP-3 + BMDM	$\frac{{10\; {\varvec{m}}{\varvec{g}}\cdot {\varvec{L}}}^{-1}}{{4.97\; {\varvec{m}}{\varvec{g}}\cdot {\varvec{L}}}^{-1}}$+$\frac{{10\; {\varvec{m}}{\varvec{g}}\cdot {\varvec{L}}}^{-1}}{2.58 {{\varvec{m}}{\varvec{g}}\cdot {\varvec{L}}}^{-1}}$	5.89	Antagonism
4-MBC + EHMC	$\frac{{10\; {\varvec{m}}{\varvec{g}}\cdot {\varvec{L}}}^{-1}}{{15.44 {\varvec{m}}{\varvec{g}}\cdot {\varvec{L}}}^{-1}}$+$\frac{{10\; {\varvec{m}}{\varvec{g}}\cdot {\varvec{L}}}^{-1}}{1.38 {{\varvec{m}}{\varvec{g}}\cdot {\varvec{L}}}^{-1}}$	7.90	Antagonism
BP-1 + BP-2 + BP-3	$\frac{{10\; {\varvec{m}}{\varvec{g}}\cdot {\varvec{L}}}^{-1}}{{9.47 {\varvec{m}}{\varvec{g}}\cdot {\varvec{L}}}^{-1}}$+ $\frac{{10\; {\varvec{m}}{\varvec{g}}\cdot {\varvec{L}}}^{-1}}{{4.52 {\varvec{m}}{\varvec{g}}\cdot {\varvec{L}}}^{-1}}$ + $\frac{{10\; {\varvec{m}}{\varvec{g}}\cdot {\varvec{L}}}^{-1}}{{4.97\; {\varvec{m}}{\varvec{g}}\cdot {\varvec{L}}}^{-1}}$	5.28	Antagonism
BP-2 + BMDM + EHMC	$\frac{{10\; {\varvec{m}}{\varvec{g}}\cdot {\varvec{L}}}^{-1}}{{9.52 {\varvec{m}}{\varvec{g}}\cdot {\varvec{L}}}^{-1}}$+ $\frac{{10\; {\varvec{m}}{\varvec{g}}\cdot {\varvec{L}}}^{-1}}{{2.58 {\varvec{m}}{\varvec{g}}\cdot {\varvec{L}}}^{-1}}$ + $\frac{{10\; {\varvec{m}}{\varvec{g}}\cdot {\varvec{L}}}^{-1}}{{1.38 {\varvec{m}}{\varvec{g}}\cdot {\varvec{L}}}^{-1}}$	13.31	Antagonism
BP-1 + BP-3 + EHMC	$\frac{{10\; {\varvec{m}}{\varvec{g}}\cdot {\varvec{L}}}^{-1}}{{9.47 {\varvec{m}}{\varvec{g}}\cdot {\varvec{L}}}^{-1}}$+ $\frac{{10\; {\varvec{m}}{\varvec{g}}\cdot {\varvec{L}}}^{-1}}{{4.97\; {\varvec{m}}{\varvec{g}}\cdot {\varvec{L}}}^{-1}}$ + $\frac{{10\; {\varvec{m}}{\varvec{g}}\cdot {\varvec{L}}}^{-1}}{{1.38 {\varvec{m}}{\varvec{g}}\cdot {\varvec{L}}}^{-1}}$	10.32	Antagonism
BP-2 + BP-3 + EHMC	$\frac{{10\; {\varvec{m}}{\varvec{g}}\cdot {\varvec{L}}}^{-1}}{{4.52 {\varvec{m}}{\varvec{g}}\cdot {\varvec{L}}}^{-1}}$+ $\frac{{10\; {\varvec{m}}{\varvec{g}}\cdot {\varvec{L}}}^{-1}}{{4.97\; {\varvec{m}}{\varvec{g}}\cdot {\varvec{L}}}^{-1}}$ + $\frac{{10\; {\varvec{m}}{\varvec{g}}\cdot {\varvec{L}}}^{-1}}{{1.38 {\varvec{m}}{\varvec{g}}\cdot {\varvec{L}}}^{-1}}$	9.47	Antagonism
BP-2 + BP-3 + BMDM	$\frac{{10\; {\varvec{m}}{\varvec{g}}\cdot {\varvec{L}}}^{-1}}{{4.52 {\varvec{m}}{\varvec{g}}\cdot {\varvec{L}}}^{-1}}$+ $\frac{{10\; {\varvec{m}}{\varvec{g}}\cdot {\varvec{L}}}^{-1}}{{4.97\; {\varvec{m}}{\varvec{g}}\cdot {\varvec{L}}}^{-1}}$ + $\frac{{10\; {\varvec{m}}{\varvec{g}}\cdot {\varvec{L}}}^{-1}}{{2.58 {\varvec{m}}{\varvec{g}}\cdot {\varvec{L}}}^{-1}}$	8.10	Antagonism
BP-2 + EHS + HMS	Not calculated	Not calculated	Not estimated

**Table 4:**
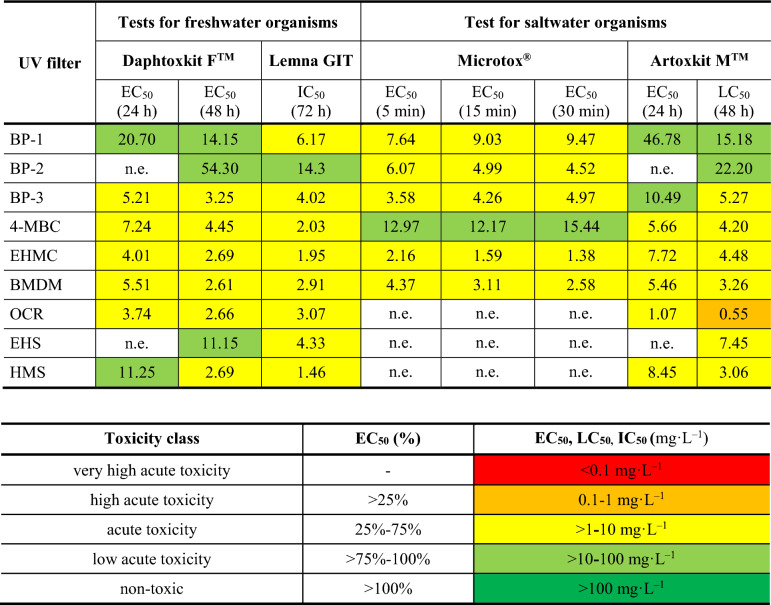
UV filters toxicity class summary according to freshwater or saltwater organism used in ecotoxicological test (n.e.—not estimated)

Based on calculated TU_mix_ values, antagonistic interactions between UV filters were dominantly predicted.

## Discussion

### Marine bacteria luminescence inhibition

After 30 min exposition, the most toxic were EHMC (1.38 mg L^–1^) and BMDM (2.58 mg L^–1^). In both cases, an increase in toxic effects over time was observed. A similar EHMC toxic concentration observed for comparable exposition time was presented by others: 1.25 mg L^–1^ (15 min) [73] and 1.06 mg L^–1^ (30 min) [74]. The significant elongation of the exposition time was correlated with an increase in toxicity (0.4 mg L^–1^/180 min) in the study of Gackowska et al. [78]. In general, reported results are in agreement with recent observations and confirm that the toxicity of EHMC to marine bacteria increases with the extension of the exposition time. However, as indicated in Figs. 1 and 2 for BP-1, BP-3, and 4-MBC an elongation of the exposure time decreased the toxic effect. It suggests that marine bacteria can adapt to the presence of the stressor and in the case of BP-3 it even creates better conditions for metabolism.

Among the benzophenone-type UV filters, the most toxic after 5 min exposition was BP-3 (3.58 mg L^–1^), however with the extension of exposition time to 30 min, BP-2 was slightly more toxic (4.52 mg L^–1^) than BP-3 (4.97 mg L.^–1^). A similar dependence was observed in the test with *Photobacterium phosphoreum* conducted by Liu et al. [74]. However, in the referenced study, EC_50_ values for BP-1 (14.21 mg L^–1^), BP-2 (8.24 mg L^–1^), and BP-3 (14.27 mg L^–1^) were higher. The tendency observed by Liu et al. [75] is probably due to the use of a different strain of luminescent marine bacteria.

In general, tested organic UV filters in the test with marine bacteria *Aliivibrio fischeri* were classified as chemicals of high acute toxicity (EHMS), acute toxicity (BP-1, BP-2, BP-3, and BMDM), and low acute toxicity (4-MBC). EC_50_ values were not estimated for OCR, EHS, and HMS. A similar observation for OCR was reported by Sanches et al. [74].

Organic UV filters are toxic to various strains of marine bacteria, however, the mechanism of toxicity is not fully known due to inconclusive results. Inhibition of bacteria luminescence occurs as a result of the reaction between the active center of organic pollutants and enzymes participating in the light reaction [38, 77]. The strength of the toxic reaction probably depends on physicochemical properties such as octanol–water partition coefficients (Log K_ow_) and the presence of electron donor substituents [74]. The lipophilic character of organic UV filters should facilitate absorption, accumulation, and inter-surface transmission across the bacterial cell membrane, however, Mayer and Reichenberg [94] suggest that compounds with Log K_ow_ > 6 require longer exposition time to express their toxicity. It can be seen, that the current results do not support the above-presented hypothesis. Based on the results displayed in Fig. 2, it can be observed that the toxicity of OCR, HMS, and EHS decreases over exposition time. Additionally, it was observed, that for OCR after 30 min exposition, the bioluminescence of marine bacteria increased. The reversal of the response between toxicity and stimulation after 30 min exposition for OCR was confirmed by others [95]. The lack of intensive luminescence inhibition after exposition to the UV filters characterized by Log K_ow_ > 6, such as OCR (Log K_ow_ = 6.88), EHS (Log K_ow_ = 6.36), and HMS (Log K_ow_ = 6.34), does not unequivocally mean that these compounds are non-toxic [100]. The low acute toxicity of EHS, HMS, and OCR against *Aliivibrio fischeri* is probably linked to their limited absorption and reduced chemical activity for bacteria cells that suggest the Log K_ow_and the presence of electron donor substituents are not the only factors responsible for the toxic response. An analysis of the structural composition of molecules suggests that both lipophilic ability and the specific type of substituents may play a crucial role in triggering the toxic response. UV filters characterized by the Log K_ow_ in the range between 2.78 and 3.79 with an abundance of the hydroxyl group (BP-1, BP-2, BP-3) trigger strong luminescence inhibition. In the case of molecules substituted by one hydroxyl group (BP-1, BP-3) luminescence inhibition slightly decreases over time, while in the case of BP-2 with 4 substituents of that type, it increases over time. The dependency between the abundance of hydroxyl groups and the intensity of the toxic effect was suggested by Liu et al. [75]. Liu et al. [75] reported that it may be related to the structural simplicity of bacteria which are not capable to react homeostatically to the greater reactivities of polar compounds.

Based on the results obtained in the Microtox® test, it can be concluded, that six of nine tested organic UV filters are toxic to marine bacteria *Aliivibrio fischeri*. An experiment confirms that a regular release of organic UV filters into the environment may harm the bacterioplankton of coastal areas that play a key role in major biogeochemical cycles and self–purification processes in marine waters and beaches [94]. Fortunately, water exchange with the open sea, waves, sea currents as well as dominantly seasonal UV filter release limits risk to the marine environment.

### Marine crustacean mortality and freshwater crustacean immobilization

Current results are similar to those presented by Thorel et al. [41]. In referenced study, after 48 h exposure, LC_50_ values were 0.60 mg L^–1^, 1.84 mg L^–1^, and 2.36 mg L^–1^ for OCR, BMDM, and HMS, respectively. On the other hand de Paula et al. [68] found the lowest LC_50_ concentrations for EHMC (0.37 mg L^–1^), followed by BMDM (2.22 mg L^–1^) and OCR (2.97 mg L^–1^). de Paula et al. [68] and Kudlek and Dudziak [81] observed toxic concentrations of BP-3 in the range of 2.0–2.53 mg L^–1^. This range is around 2 times lower in comparison to the result obtained in a current experiment. Based on identified EC_50_ and LC_50_ values in the test with *Artemia franciscana*, six of nine organic UV filters were classified as acutely toxic. OCR was classified as high acutely toxic, while BP-1 and BP-2 were the least toxic and classified as low acutely toxic.

Exposure crustaceans to hydrophobic UV filters resulted in a behavioral change manifested by reverse motion. The reduced phototaxic response may be related to neurotoxic effects linked with the disruption of the optimal enzyme acetylcholinesterase (AChE) level leading to disruption of nervous function [96]. Such disturbances have been observed after exposure of fish larvae to some UV filters. Sandoval-Gío et al. [97] reported an increase in AChE gene expression and a decrease in AChE activity after exposure of zebrafish larvae to BP-3 without significant behavioral changes. On the other hand, Araújo et al. [46] observed an increase in AChE after exposure of flatfish (*Solea senegalensis*) to 4-MBC linked with behavioral changes manifested as impairment in swimming behavior response to light stimulus. Significant inhibition of the locomotor activity linked with AChE inhibition was observed after exposure of zebrafish larvae to 4-MBC [98] and BMDM [99].

The toxic effect of various intensities of nine tested organic UV filters was observed in tests with saline and freshwater crustaceans. Current results indicate that UV filters’ toxicity increases over time. In both crustacean tests, OCR and BMDM were the most toxic. In contrast to the Microtox® test, the least toxic for crustaceans were BP-1 and BP-2. Among the benzophenone-type UV filters, EC_50_ and LC_50_ values after 48 h exposition of crustaceans can be ranked in the following, descending order: BP-3 > BP-1 > BP-2. Moreover, a higher resistance of *Daphnia magna* than *Artemia franciscana* against BP-2 was discovered. Current results are in agreement with results presented by Liu et al. [75] both in terms of organisms’ sensitivity as well as in terms of descending toxicity of benzophenones. In referenced study, freshwater crustaceans *Daphnia magna* were also less sensitive to BP-1 and BP-2 in comparison to the marine bacteria *Photobacterium phosphoreum*. It appears that the benzophenone derivatives mechanism of toxicity against crustaceans depends on the Log K_ow_ value and water solubility. BP-2 is characterized by the lowest Log K_ow_ value and contains 4 hydroxyl substituents in comparison to BP-1 and BP-3 which are substituted by a single hydroxyl. The higher number of hydroxyl substituents facilitates the creation of hydrogen bonds and hence contributes to BP-2 higher solubility in water and the lowest toxic effect. A similar tendency was observed by others [82, 87, 107]. Crustaceans’ exposition to hydrophobic UV filters resulted in a behavioral change manifested by reverse motion. Behavioral disorders, observed also by Boyd et al. [67], probably depend on reduced phototactic response due to impairment of the physical ability to react or reduced sensitivity to light as described in the saline crustaceans test. As a consequence of any behavioral disorders, limited sensitivity to light, in particular, may affect the elimination of crustaceans from the population [66, 99]. Correct detection and response to light stimuli are essential for moving in the water columns, getting food, and escaping predators.

### Aquatic plant *Lemna minor* growth inhibition

Based on calculated IC_50_ values eight of nine tested UV filters were classified as compounds of acute toxicity against *Lemna minor*, while BP-2 was classified as a molecule of low acute toxicity. Comparison with other, recent studies revealed, that Kudlek and Dudziak [81] indicated growth inhibition of *Lemna minor* after exposition to BP-3 at a concentration of 1 mg L^–1^, which is around 4 times higher than in current research. *Lemna minor’s* growth inhibition is associated with negative reactions in the photosynthesis systems of plants. Based on observations of the toxic effects of UV filters on green algae, Mao et al. [49] suggested that the effect depends on the Log K_OW_ value of a given compound. It seems that Log K_ow_ is one of the most important factors that trigger toxicity against various groups of organisms used in modern ecotoxicology. UV filters characterized by the higher value of Log K_ow_ are more easily uptaken by plants and pass through the double membrane. A similar explanation was proposed by Zhong et al. [101], who studied the toxic effects of the exposition of green cucumber (*Cucumis sativus* L.) to BP-3, BMDM, OCR, and EHMC. In the referenced study researchers observed that the assimilation of lipophilic UV filters may inhibit photosynthetic electron transport, limiting the production of energy transfer compounds. It triggered the production and accumulation of reactive oxygen radicals that damaged cell membranes, and photosynthetic apparatus and limited the assimilation of CO_2_. The results obtained in the current research fill the gap regarding the toxicity of organic UV filters against *Lemna minor.*

Based on data presented in Table 4 it seems that UV filters create a higher environmental risk to freshwater organisms. In the majority of cases observed effects and successfully estimated EC_50_/IC_50_ values allowed the classification of tested filters as acutely toxic. Limited water exchange and constant UV filters release from insufficiently treated sewage cause serious threats to freshwater organisms. Even if no physiological disruptions are detected in acutely exposed organisms delayed mortality can take place [67]. Contrarily, in seawater usually higher self-purification and dilution due to waves and sea currents as well as only seasonal UV filter releases take place. Moreover, none of the toxic effects was observed for three out of nine UV filters. Additionally, for some of them, an adapting ability was observed in the test with marine bacteria.

### Toxicity of mixtures

Organisms inhabiting various environmental compartments are exposed to a variety of UV filter mixtures released to the environment. In general, so far little is known about the interactions of chemicals in mixtures. On the one hand, it is commonly found that mixed toxic chemicals result in synergistic or additive effects, however, on the other hand, there are also some pieces of evidence about antagonistic interactions between UV filters [102–104]. Based on the results presented in Table 3 it can be summarized that the calculated total TU value of 16 out of 20 binary and ternary mixtures was greater than 1, indicating that the toxicity of mixtures on *Allivibrio fisheri* was antagonistic. According to references [105], an antagonistic effect of mixtures with benzophenones as a major component is probably related to their structural similarity manifested in the number and locations of hydroxyl groups, as is in the case of BP-1, BP-2, and BP-3. The competition they pose in the active parts of the cell surface and the metabolic system due to their structural similarity may be responsible for their antagonistic effect. The final toxicity of the benzophenone-based mixture may be due to the differences in relative potencies of single compounds, however, its explanation is not a trivial task since it is highly dependent on the initial concentration of components [44, 45]. Similarly, as in the current study, some antagonism was observed in binary mixtures of UV filters with BP-1 at the equipotent mixture level [45] implying that BP-1, and probably other benzophenones, possesses higher relative potency, as a factor, which can trigger antagonistic action when mixed with other UV filters. Some pieces of evidence concerning the difference in the relative potency of filters as a factor that triggers antagonistic effects in mixtures were also found for EHMC, BMDM, and OCR. Park et al. [44] observed that mixtures of different concentrations of EHMC, BMDM, and OCR triggered lower toxic effects than exposition to single UV filters to *Daphnia magna*. Unfortunately, a complete reduction of toxic effects in the mixtures could not be fully achievable due to the difficulty to estimate the interaction of other filters. Analyzing the overall toxic effects of binary or ternary mixtures we suspect that the lipophilic components, such as OCR, HMS, and EHS can promote penetration of hydrophilic filters into the bacterial cell causing a toxic effect. In general, current results are in agreement with other studies concerning interactions between components of mixtures, except for the combination of BP-3 + EHMS that showed a synergistic effect against *Daphnia magna* [105], Zhang et al. [106] found that a mixture of benzophenone-6 (BP-6), benzophenone-8 (BP-8), and 4-MBC showed antagonistic toxicity against four *Salmonella typhimurium* strains in the Ames test. Other tests with organisms from higher trophic levels confirmed the possibility of antagonistic interaction between toxic compounds in mixtures. Antagonistic interaction of binary and ternary mixtures of 4,4′-dihydroxybenzophenone (4,4′-HBP), 2,4,4′-trihydroxybenzophenone (2,4,4′-HBP), and 4-MBC that triggered toxicity against *Chlorella vulgaris* was observed by Han et al. [103]. Du et al. [102] have shown that the toxic effects of the mixture composed of BP-3 and benzophenone-4 against *Chlorella vulgaris*, *Daphnia magna*, and *Danio rerio* were also antagonistic. However, some other data suggest that the interactions between UV filters in mixtures could lead to additive or synergic endpoints. In the study referenced above one combination of a binary mixture composed of 4,4′-HBP and 4-MBC exhibited an additive toxic effect [103]. Cahova et al. [47] indicated higher toxicity after the exposition of *Danio reiro* embryos to a mixture of 4-MBC and OCR, a mixture of BP-3, EHMC, and 2-phenylbenzimidazole-5-sulfonic acid (PBSA) and the combination of all five UV filters than in case of exposition to a single solution. Based on the results of hatching rate and mortality, the authors concluded that mixtures of tested UV filters showed an additive toxic effect. Having in mind that UV filters are commonly used as mixtures and in the form of multicomponent solutions are released to aquatic environments, it is of high importance to analyze their combined toxic effect of them. Current results partially fill an existing gap in the assessment of joined toxic effects of various mixtures against standardized organisms, however, to precisely interpret the reason why specific mixtures exhibit the antagonistic or synergic effect in a given concentration against a given bioindicator, more work needs to be done, and more sophisticated tools, such as quantitative structure–activity relationship models coupled with an analysis of gene transcriptional activity need to be used. For example, some authors suggest that 4MBC, BP-3, and OMC may have antagonist effects on EcR gene transcription and a synergistic effect on hsp70 gene activation [86].

Toxicity assessment using single UV filters confirmed toxic or harmful effects for standardized organisms using commonly in modern ecotoxicological biotests and provided relevant data on the toxicity of nine UV filters. Toxic concentrations estimated for nine organic UV filters were in the range between 0.55 and 54.30 mg L^–1^. OCR was one of the most toxic UV filters in the test with *Artemia franciscana*, *Lemna minor,* and *Daphnia magna*. Surprisingly, OCR against marine bacteria *Aliivibrio fischeri* was classified as non-toxic. Equivocal responses against different test organisms confirm that the use of biotest sets brings more comprehensive information on sensitivity and environmental threats. The obtained results indicate that UV filters can pose a threat at every level of the food web. Toxicity assessment using binary and ternary mixtures indicated prevailing domination of high acute toxicity or acute toxicity effects against *Aliivibrio fischeri*. Surprisingly, the mixtures containing non-toxic UV filters (OCR, EHS, HMS) combined with BP-2 were highly acutely toxic. Perhaps lipophilic UV filters may facilitate penetration of hydrophilic UV filters into the cell, causing a toxic effect. The predicted mechanism of toxic interactions between tested xenobiotics was mainly antagonistic.

The toxic concentration values for a single solution were estimated at levels higher than concentration values determined in many aquatic environmental compartments [4, 10, 16, 17, 21, 25, 107]. However, taking into account the widespread use of UV filters and their constant release into the aquatic environment, it can be predicted that environmental balance can be knocked out of the state of equilibrium shortly. As a consequence, environmental concentrations of many non-biodegradable compounds may increase significantly and pose a real threat to living organisms. Moreover, commonly aquatic organisms are exposed to mixtures of UV filters coupled with a variety of other pollutants. Because of this, it is necessary to conduct further ecotoxicological research with UV filter mixtures to better understand the mechanisms of toxicity and interactions.

## Data Availability

The datasets generated during the current study are available from the corresponding author on request.
